# Silencing ZIC5 suppresses glycolysis and promotes disulfidptosis in lung adenocarcinoma cells

**DOI:** 10.1080/15384047.2025.2501780

**Published:** 2025-05-14

**Authors:** Cimei Zeng, Denggao Huang, Lei Wang, Haimei Liang, Ximiao Ma

**Affiliations:** a Department of Respiratory and Critical Care Medicine, Affiliated Haikou Hospital of Xiangya Medical College, Central South University, Haikou, Hainan, China; b Central Laboratory, Affiliated Haikou Hospital of Xiangya Medical College, Central South University, Haikou, Hainan, China; c Thoracic Surgery, Affiliated Haikou Hospital of Xiangya Medical College, Central South University, Haikou, Hainan, China

**Keywords:** Lung adenocarcinoma, ZIC5, glycolysis, glucose metabolism, disulfidptosis

## Abstract

**Objective:**

This study aims to explore the effects of silencing Zic family member 5 (ZIC5) on glucose metabolism and disulfidptosis in lung adenocarcinoma (LUAD) cells.

**Methods:**

Data from The Cancer Genome Atlas (TCGA) was used to analyze ZIC5 expression in LUAD and its association with patient outcomes. ZIC5 was silenced in A549 and H1299 cells using siRNA. The expression of ZIC5 mRNA and protein was assessed by qRT-PCR and Western blot. Cell proliferation was evaluated through CCK-8 and 5-ethynyl-2’-deoxyuridine (EdU) assays, while glucose uptake, lactate production, and ATP levels were measured to assess energy metabolism. Seahorse XF analysis was used to evaluate extracellular acidification rate (ECAR) and oxygen consumption rate (OCR). Disulfidptosis was assessed through NADP^+^/NADPH ratio, glutathione (GSH) content, GSSG/GSH ratio, and immunofluorescence staining.

**Results:**

ZIC5 is highly expressed in LUAD and is associated with poor patient prognosis. Silencing ZIC5 significantly reduced its mRNA and protein levels in A549 and H1299 cells, markedly inhibited cell proliferation, and led to significant decreases in glucose uptake, lactate production, ATP levels, ECAR, and OCR. Additionally, silencing ZIC5 resulted in an increased NADP^+^/NADPH ratio, decreased GSH levels, and a reduced GSSG/GSH ratio, alongside classic disulfidptosis features.

**Conclusion:**

ZIC5 plays a crucial role in promoting LUAD cell proliferation and energy metabolism while inhibiting disulfidptosis. Silencing ZIC5 markedly suppresses these processes, indicating its potential as a therapeutic target in LUAD.

## Introduction

Lung adenocarcinoma (LUAD) is one of the predominant subtypes of non-small cell lung cancer (NSCLC), accounting for 50% of all lung cancer cases.^1^ The etiological factors of LUAD involve environmental factors like smoking and air pollution, as well as genetic mutations such as EGFR and ALK, particularly in younger patients.^2^ While LUAD is a leading cause of cancer mortality globally, its incidence in younger populations is also notable, despite its prevalence in the elderly.^3^ LUAD shows high heterogeneity; early-stage prognosis is good, but invasive adenocarcinoma has poor outcomes.^4^ Although targeted and immunotherapies are effective, they are limited in treating specific LUAD subtypes, early identification of high-risk LUAD patients and the development of personalized treatments remain significant challenges.^5^ Abnormal gene expression plays a crucial role in the diagnosis, prognosis, and pathogenesis of LUAD.^6–8^

Zic family member 5 (ZIC5), part of the zinc finger protein family, has recently been linked to tumor etiology and metastasis.^9^ Studies have shown that ZIC5 promotes tumor growth and metastasis by regulating cell proliferation, migration, and stemness, and is closely associated with cancer prognosis.^10,11^ Additionally, ZIC5 is involved in regulating metabolic reprogramming, influencing the metabolic state of tumor cells, and thus providing a potential target for cancer therapy.^12^ Dong et al. found that ZIC5 is upregulated in lung cancer, indicating its potential as a prognostic biomarker.^11^ However, the specific role and mechanisms of ZIC5 in LUAD remain unclear, necessitating further exploration. Metabolic reprogramming, a hallmark of cancer cells, notably the Warburg effect, enhances glycolytic pathways to meet the energy and metabolic demands of rapid proliferation.^13^ Disruptions in redox balance, particularly through glutathione (GSH) metabolism, are common in tumor cells.^14^ Research indicates that oxidative stress is closely associated with apoptosis, necrosis, and other forms of programmed cell death.^15^ Metabolic cell death, a form of regulated cell death closely linked to metabolic stress, plays a crucial role in the metabolic treatment of cancer, offering potential targets for novel therapeutic strategies.^16^ Among these mechanisms, disulfidptosis, a newly discovered mode of cell death, has garnered significant attention. Intracellular disulfide bonds, when abnormally aggregating with the actin cytoskeleton proteins, lead to actin clustering and subsequent disruption of the cellular structure, culminating in disulfidptosis.^17^ Recent findings have highlighted its significant role in modulating LUAD.^18^

Therefore, this study aims to investigate the role of ZIC5 in LUAD, focusing on its regulation of glycolysis and disulfidptosis to uncover new therapeutic targets.

## Materials and methods

### Bioinformatics analysis

To investigate the expression of the ZIC5 gene in LUAD and its relationship with patient prognosis, RNA sequencing data for LUAD patients and corresponding normal tissues were initially retrieved from the Cancer Genome Atlas (TCGA) database (https://portal.gdc.cancer.gov/., TCGA-LUAD, version 202208). LUAD patient samples in the TCGA database must meet the following criteria: confirmed diagnosis of LUAD with no prior neoadjuvant therapy, tumor tissue samples containing ≥ 80% tumor cells, and availability of matched normal tissue. Samples must have complete clinical information and ensure RNA and DNA quality meet sequencing requirements (RIN >7). Differential expression analysis was conducted using the edgeR package (version 3.28.1, https://bioconductor.org/packages/release/bioc/html/edgeR.html.) in R, comparing the expression levels of ZIC5 in normal tissues (*n* = 59) and tumor tissues (*n* = 539). ZIC5 expression was categorized into high and low expression groups based on the median expression value. Samples with ZIC5 expression higher than the median were classified as high expression, and samples with ZIC5 expression lower than or equal to the median were classified as low expression. To validate the association between ZIC5 expression levels and patient survival, Kaplan-Meier survival curve analysis was performed using the survival package (version 2.44–1.1, https://cran.r-project.org/web/packages/survival/index.html), and the impact of high versus low ZIC5 expression on survival was assessed using the Cox proportional hazards model. Additionally, the gene expression matrix and correlation coefficients (calculated using the Pearson method, with a threshold set at 0.05) were used to explore the correlations between ZIC5 and several glycolysis-related genes (such as HIF1A, EPO, LDHA, etc.). Correlation analysis was implemented in R using the pheatmap package (version 1.0.12, https://cran.r-project.org/web/packages/pheatmap/index.html), generating expression correlation heatmaps to visualize the associations between these genes. All statistical analyses were performed in the R environment, ensuring the statistical significance and reproducibility of the experimental results.

### Cell culture

The A549 cell line carries KRAS mutations and TP53 wild-type characteristics and is widely used to model the molecular mechanisms of KRAS mutations in lung adenocarcinoma.^19^ The H1299 cell line, on the other hand, is a TP53-null lung adenocarcinoma cell line commonly used to study the impact of TP53 loss on tumorigenesis and progression.^20^ Both cell lines serve as models with genetic mutation features similar to those observed in the TCGA-LUAD dataset, providing a good representation of the genetic variation background in actual clinical samples. A549 (Procell, CL-0016) and H1299 cells (Procell, CL-0165) were cultured in F-12K medium (Gibco, 21127–022) and RPMI-1640 medium (Gibco, 11875–093), respectively, with both media supplemented with 10% fetal bovine serum (FBS, Gibco, 26140–079). The cells were incubated in a cell culture incubator (Thermo Fisher Scientific, 3111) at 37℃ with 5% CO_2_ and 95% humidity. After 24 h of incubation, cells were digested with Trypsin-EDTA (Gibco, 25200–056) and seeded onto glass coverslips at a density of 2 × 10^4^ cells per coverslip, followed by an additional 24 h of incubation. Upon completion of the cell culture process, cell morphology was observed under bright-field microscopy, and GFP-labeled cells were visualized using a fluorescence microscope (Nikon, Ti2).

### Green fluorescent protein (GFP)-labeled cells

A549 and H1299 cells were transfected with a GFP expression plasmid using Lipofectamine 2000 transfection reagent (Invitrogen, 11668019) when they reached 50%–70% confluence. The GFP plasmid and transfection reagent were mixed at a 1:2 ratio and incubated for 10 min at room temperature to form transfection complexes. The complexes were then added to the cells and incubated for 4–6 h. Following transfection, the medium was replaced with complete culture medium containing 10% FBS, and the cells were incubated for an additional 24–48 h to ensure GFP expression. After transfection, GFP fluorescence was observed using a fluorescence microscope (Nikon, Ti2) under 488 nm excitation light, cells morphology and transfection efficiency were preliminarily evaluated based on GFP fluorescence distribution.

### Vector construction and transfection

The GeneBank ID of ZIC5 is NM_033132.5. si-ZIC5 and NC-si lentiviruses were synthesized by Guangzhou Anernor Biotechnology Co., Ltd. The sequences for si-ZIC5 #1, si-ZIC5 #2, and NC-si were as follows: si-ZIC5 #1: 5’-CGCCCAAUACAUACAUGUUUU-3’; si-ZIC5 #2: 5’-GCCCAAUACAUACAUGUUUUU-3’; NC-si: 5’-UUCUCCGAACGAGUCACGUTT-3’. A549 and H1299 cells were seeded at a density of 2 × 10^4^ cells per well in 12-well plates (Corning, 3513). According to the manufacturer’s instructions, siRNA and Lipofectamine 3000 (Invitrogen, L3000015) were mixed with Opti-MEM medium (Gibco, 31985–070) to form transfection complexes. The transfection complexes were then added to the cell culture medium, gently mixed, and incubated at 37℃ for 6 h before being replaced with fresh medium.

### qRT-PCR

Total RNA was extracted using Trizol reagent (Tiangen). Each 1.5 mL EP tube received 500 μL of Trizol, followed by 100 μL of chloroform (Fuzhou Guangbo Adhesive Industry). After shaking and letting it stand for 5 min, the mixture was centrifuged at 16,097 ×g for 10 min at 4℃. The upper clear aqueous phase was transferred to a clean 1.5 mL EP tube, mixed with an equal volume of isopropanol (Sinopharm Chemical Reagent Co., Ltd.), and allowed to stand for 10 min. This was followed by centrifugation at 16,097 ×g for 10 min at 4℃. The supernatant was discarded, and the RNA pellet was washed with 1 mL of 75% ethanol, centrifuged at 5478 ×g for 5 min at 4℃. After removing the supernatant, the RNA was air-dried at room temperature for 10 min and dissolved in 25 μL of DEPC-treated water (Sigma, 40718). The RNA was stored at −80℃. RNA concentration was measured using a micro-spectrophotometer (Hangzhou Allsheng Instruments Co., Ltd., Nano-200). The reverse transcription reaction was performed in a 20 μL system, containing 3 μg of total RNA, 2.5 μL of Oligo(dT) primer (Quanshijin), 12.5 μL of DEPC-treated water, 4 μL of RT buffer, 2 μL of dNTPs (Thermo, GD1102), 1 μL of RevertAid Reverse Transcriptase (Thermo, EP0441), and 0.5 μL of RNase inhibitor (Thermo, E00381). The reaction conditions were 70℃ for 5 min, 42℃ for 60 min, and 70℃ for 10 min. The qRT-PCR reaction was performed in a 10 μL system, containing 5 μL of SYBR Green I mix (Quanshijin, AQ601–04), 0.3 μL of forward primer (10 μM, Sangon Biotech), 0.3 μL of reverse primer (10 μM, Sangon Biotech), 0.2 μL of cDNA, and sterile distilled water up to 10 μL. The amplification program was set to 94℃ for 10 min, followed by 40 cycles of 95℃ for 5 s, 60℃ for 15 s, and 72℃ for 10 s. The melting curve program was 60℃ for 8 s and 95℃ for 8 s. A real-time fluorescent quantitative PCR instrument (ABI, Q1) was used to detect ZIC5 mRNA expression levels which were analyzed using the 2^−ΔΔCt^ method, with GAPDH as the internal reference gene. The sequences for the primers were as follows: ZIC5-forward: 5’-AAACTTTCGGCACCATGCAC-3’; ZIC5-reverse: 5’-CGGGGACTTGCAGTGAATCT-3’; GAPDH-forward: 5’-AGCCCAAGATGCCCTTCAGT-3’; GAPDH-reverse: 5’-CCGTGTTCCTACCCCCAATG-3’

### Western blot

The collected cells were washed with 1 mL of physiological saline and transferred to a 1.5 mL centrifuge tube. One mL of RIPA lysis buffer (Beyotime, P0013B) and 10 μL of PMSF (100 mM) were added, mixed thoroughly, and lysed on ice for 30 min. The lysate was centrifuged at 16,097 ×g for 10 min at 4℃. The supernatant was aliquoted into new centrifuge tubes for protein quantification. Protein concentration was determined using a BCA Protein Assay Kit (Beyotime, P0012). Samples were mixed with BCA working solution and incubated at 37℃ for 30 min. After cooling to room temperature, the absorbance at 562 nm was measured using a microplate reader (BioTek, ELX800). Protein samples were mixed with 5× loading buffer at a 4:1 ratio and boiled for 10 min. After cooling, samples were subjected to SDS-PAGE. A 9% resolving gel and a 5% stacking gel were prepared, with 20 μg of protein loaded per lane. Electrophoresis conditions were 80 V for 40 min for the stacking gel and 120 V for 30 min for the resolving gel. Proteins were transferred to PVDF membranes (Merck Millipore, IPVH00010) at a constant voltage of 100 V. After transfer, PVDF membranes were blocked with 5% BSA-PBST at room temperature for 1 h. The membranes were then incubated overnight at 4℃ with primary antibodies diluted in 5% BSA-PBST: anti-ZIC5 antibody (Thermo Fisher, PA5–142027, 1 µg/mL) and anti-GAPDH antibody (Proteintech, 60004–1-Ig, 1:5000). The next day, the membranes were washed five times in PBST, each for 6 min. Secondary antibody incubation was performed with HRP-conjugated anti-rabbit IgG (KPL, 074–1506, 1:5000) and anti-mouse IgG (KPL, 074–1807, 1:5000) at room temperature for 1 h. The membranes were washed again five times in PBST, each for 6 min. Detection was carried out using ECL reagents (Beyotime, P0018S). Solutions A and B were mixed at a 1:1 ratio and applied to the membranes. Signals were visualized using a chemiluminescence imaging system (Shanghai Qinchang, ClinxChemiScope 6000). Appropriate exposure times were set, and images were saved and exported. Finally, the grayscale values of protein bands were quantified using Image J software (National Institutes of Health).

### CCK-8

After cell recovery, cells were cultured in DMEM medium (Hyclone, SH30022.01) containing 10% fetal bovine serum (Gibco, A5670701) and 1% penicillin-streptomycin at 37℃ in a 5% CO_2_ incubator (Thermo Fisher, 3111) until reaching the logarithmic growth phase. Cells were seeded at a density of 5000 cells per well in 96-well plates. Once stabilized, CCK-8 assays were performed on days 1, 2, 3, 4, and 5. At each time point, 1/10 volume of CCK-8 reagent (Quanshijin, FC101–03) was added to each well and incubated at 37°C in a 5% CO_2_ incubator for 1 h. Absorbance (OD) at 450 nm was measured using a microplate reader (BioTek, ELx800). A cell growth curve was plotted with time on the x-axis and OD values on the y-axis to evaluate cell proliferation. For calculating cell death rates, cells were seeded in 6-well plates at a density of 3 × 10^5^ cells per well. Different treatments were added: DMSO (Sigma, D2650, 0.1% v/v), Z-VAD (Sigma 627610, 20 μM), Ferrostatin-1 (Sigma, SML0583, 1 μM), TCEP (Sigma, C4706, 100 μM). After 24 h of incubation, cells were processed using the CCK-8 method as described above. Cell death rate (%) was calculated using the following formula: Cell death rate (%) = (1 - OD value of treatment group/OD value of control group) × 100.

### 5-ethynyl-2’-deoxyuridine (EdU)

Cells were digested with 0.25% trypsin (Invitrogen) and seeded at a density of 5000 cells per well in 96-well plates. EdU (10 μM, Invitrogen, C10337) was added, and cells were incubated at 37℃ for 2 h. After fixation, cells were stained using the Click-iT EdU Cell Proliferation Kit (Invitrogen, C10337) according to the manufacturer’s instructions, followed by counterstaining with DAPI (Sigma, D9542) for nuclei. Finally, images were captured using a fluorescence microscope (Nikon, Ti2).

### Measurement of 24 h glucose uptake, lactate production, and ATP content

Glucose uptake was measured using the Amplex® Red Glucose/Glucose Oxidase Assay Kit (Invitrogen, MP 22189). Preparation included a 10 mm Amplex® Red reagent stock solution, 1×reaction buffer, 10 U/mL horseradish peroxidase stock solution, 100 U/mL glucose oxidase stock solution, and 400 mm glucose stock solution. After 24 h of cell culture, cells were digested and resuspended in 1 mL extraction buffer. Cells were lysed by sonication on ice and centrifuged at 1200 g for 10 min at 4℃. The supernatant was used for glucose measurement. Samples (50 μL each) were added to a 96-well plate, followed by the addition of 50 μL of Amplex® Red/HRP/glucose oxidase working solution. After incubating at room temperature in the dark for 30 min, fluorescence was measured at 590 nm using a microplate reader (BioTek, ELx800) to determine glucose content. Lactate production was measured using the Lactate Assay Kit (Abcam, ab65330). After 24 h of cell culture, cells were digested and resuspended in 1 mL extraction buffer. Cells were lysed by sonication with a power of 300 W (3 s on, 7 s off) for a total of 3 min. After centrifugation at 1200 g for 10 min at 4℃, the supernatant was used for lactate measurement. For each sample, 10 μL of supernatant was mixed with 90 μL of distilled water and 60 μL of the colorimetric reagent. After reacting at 37℃ for 30 min, absorbance was measured at 570 nm using a microplate reader (BioTek, ELx800). ATP content was measured using the ATP Assay Kit (Beyotime, S0026) according to the manufacturer’s instructions. Each sample was mixed with 100 μL of ATP detection working solution and incubated at room temperature for 5 min. Then, 20 μL of sample or standard was added, quickly mixed, and relative luminescence units (RLU) were measured using a luminometer (BioTek, Synergy H1).

### Measurement of cellular extracellular acidification rate (ECAR) and oxygen consumption rate (OCR)

Cells were seeded in Seahorse XF96 microplates at a density of 2 × 10^4^ cells per well. ECAR was measured using the Seahorse XF Glycolysis Stress Test Kit (Agilent Technologies 103020–100). Glycolytic activity was monitored in real-time by sequentially adding 2-deoxy-D-glucose (2-DG, Sigma, D8375, 50 mm), and a mixture of rotenone and antimycin A (Rot/AA, Sigma, R8875/A8674, 1 μM). OCR was measured using the Seahorse XF Cell Mito Stress Test Kit (Agilent Technologies 103015–100, 1.5 μM). Mitochondrial respiration activity was monitored in real-time by adding oligomycin (Sigma, O4876, 1 μM), carbonyl cyanide-p-trifluoromethoxyphenylhydrazone (FCCP, Sigma, C2920, 1 μM), and a mixture of rotenone and antimycin A (Rot/AA).

### Measurement of cellular NADP^+^/NADPH ratio, GSH content, and GSSG/GSH ratio

The cellular NADP^+^/NADPH ratio was measured using the NADP^+^/NADPH Quantitation Kit (Sigma, MAK038). Cells were collected, and NADP^+^ and NADPH contents were extracted and measured according to the kit’s instructions. The GSH content and GSSG/GSH ratio in cells were determined using the GSH/GSSG Quantification Kit (Beyotime, S0053). The procedures followed the kit instructions, and absorbance values were read at 405 nm using a microplate reader (BioTek, ELx800).

### Flow cytometry analysis

Cell death rate was analyzed using flow cytometry to assess the effects of different treatments on cell viability. A549 and H1299 cells were treated with various compounds. The medium used was DMEM (Gibco 11965092), supplemented with 10% fetal bovine serum (FBS, Gibco 10099141) and 1% penicillin/streptomycin (Gibco 15140122). Cells were cultured in a humidified incubator at 37℃ with 5% CO_2_. Prior to treatment, cells were cultured until approximately 80% confluency and then digested using trypsin (Gibco 25300054). After digestion, cells were reseeded for further experiments. ZIC5 gene knockdown was achieved by transfecting siRNA constructs, including si-ZIC5 #1 and si-ZIC5 #2, along with the control NC-si. Transfection was carried out using Lipofectamine 2000 (Invitrogen 11668027) according to the manufacturer’s instructions. After 24 h of transfection, cells were treated with the following compounds: Z-VAD (Sigma 627610 20 μM), Ferrostatin-1 (Sigma, SML0583, 1 μM), and TCEP (Sigma, C4706, 100 μM), with DMSO (Sigma, D2650, 0.1% v/v) as the solvent. Following 24 h of treatment at 37℃, cells were analyzed for cell death using flow cytometry (BD FACSCanto II, BD Biosciences, 643012). Cells were stained with FITC-conjugated Annexin V (BD Biosciences, 556419) and propidium iodide (PI, BD Biosciences, 556463) for dual labeling, and cell death was quantified. The data were analyzed using FlowJo software (FlowJo LLC, version 10.8.1).

### Immunofluorescence staining for CD44 and actin expression

Cells were fixed and permeabilized with 0.1% Triton X-100 (Sigma, T8787), then blocked with 5% BSA (Sigma, A2153) for 1 h. The cells were incubated overnight with anti-CD44 antibody (Cell Signaling Technology, 3570S, 1:200) and anti-actin antibody (Sigma, A2066, 1:200). The next day, cells were incubated at room temperature for 1 h with Alexa Fluor 488-conjugated secondary antibody (Invitrogen, A-11008, 1:500) and Alexa Fluor 594-conjugated secondary antibody (Invitrogen, A-11012, 1:500). Nuclei were counterstained with DAPI, and images were captured using a fluorescence microscope (Nikon, Ti2).

### Statistical analysis

Statistical analysis was performed using GraphPad Prism 9 software (GraphPad Software, San Diego, CA, USA). All data were expressed as mean ± standard error of the mean (SEM). One-way analysis of variance (ANOVA) was used for comparisons between groups, with *p* < .05 considered statistically significant.

## Results

### High expression of ZIC5 in LUAD is associated with poor prognosis

**Figure 1.**
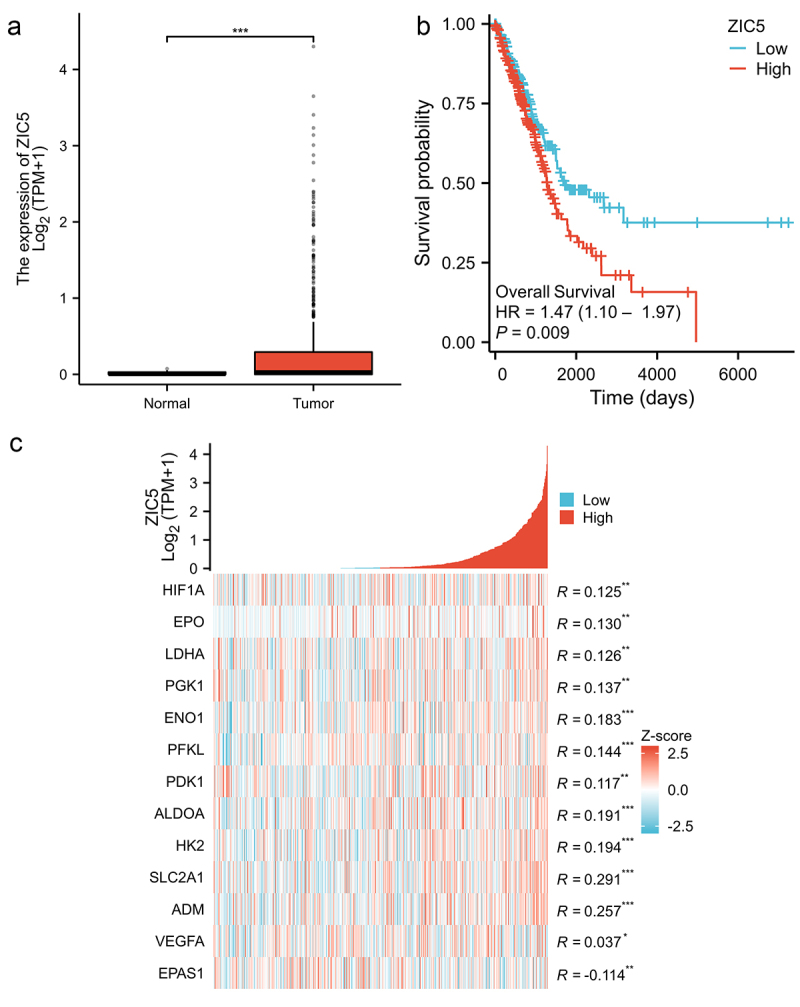
High expression of ZIC5 in LUAD is associated with poor prognosis. RNA sequencing data for LUAD patients and their corresponding normal tissues were obtained from the TCGA database. (a) Comparison of ZIC5 expression levels in normal (*n* = 59) and LUAD tissues (*n* = 539). (b) Comparison of overall survival rates in patients with different ZIC5 expression levels. (c) Heatmap showing the correlation between ZIC5 expression levels and glycolysis-related genes in tumor tissues. LUAD: lung adenocarcinoma, TCGA: the cancer genome atlas. *p < .05, **p < .01, ***p < .001.

RNA sequencing data for LUAD patients and corresponding normal tissues were obtained from The Cancer Genome Atlas (TCGA) database. Differential expression analysis was performed using the edgeR package in R. The results showed that ZIC5 expression was significantly higher in tumor tissues compared to normal tissues, the median of the Normal group is 0 (Q1 = 0, Q3 = 0.028), and the median of the Tumor group is 0.031 (Q1 = 0, Q3 = 0.292) (Figure 1a). Kaplan-Meier survival curve analysis and Cox proportional hazards regression analysis were conducted to compare the overall survival rates of patients with high and low ZIC5 expression. The results indicated that patients with high ZIC5 expression had significantly lower overall survival rates than those with low ZIC5 expression, suggesting that high ZIC5 expression is associated with poor prognosis (Figure 1b). Additionally, a heatmap was used to display the correlation between ZIC5 expression levels and multiple glycolysis-related genes (HIF1A、EPO、LDHA、PGK1、ENO1、PFKL、PDK1、ALDOA、HK2、SLC2A1、ADM、VEGFA and EPAS1) in LUAD tissues. It was found that high ZIC5 expression was positively correlated with the expression of these glycolysis genes except EPAS1 (Figure 1c). It is important to note that the correlations observed between the studied genes are modest, suggesting that while associations are present, they may not represent very strong or definitive relationships. These results reveal that ZIC5 is highly expressed in LUAD and is involved in the regulation of the glycolysis pathway, with this high expression significantly associated with poor patient prognosis.

### Significant decrease in ZIC5 mRNA and protein levels following siRNA silencing

**Figure 2.**
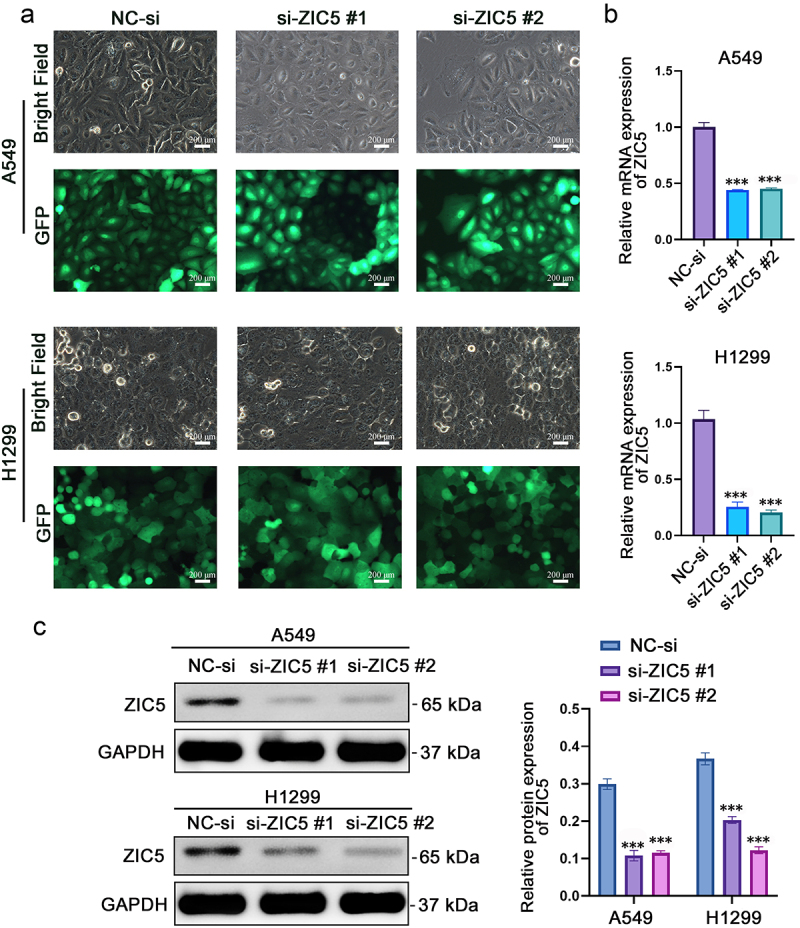
Significant decrease in ZIC5 mRNA and protein levels following siRNA silencing. (a) Observation of cell morphology changes using bright-field and GFP fluorescence microscopy. (b) qRT-PCR analysis of ZIC5 mRNA expression levels. (c) Western blot analysis of ZIC5 protein expression levels. GFP: green fluorescent protein. Compared to the NC-si group, ***p < .001.

Compared to the control group (NC-si), bright-field microscopy revealed that A549 and H1299 cells exhibited morphological changes, including deformation, shrinkage, and loss of typical epithelial characteristics, following ZIC5 silencing. GFP fluorescence microscopy was used to observe the distribution of GFP. Normal A549 and H1299 cells displayed distinct green fluorescence and uniform morphology under GFP fluorescence microscopy, consistent with expected cell morphological characteristics. In contrast, cells treated with ZIC5-targeting siRNA showed uneven distribution of GFP fluorescence (Figure 2a). This observation is in line with previous studies demonstrating that GFP fluorescence can be used as an indicator of cell viability and transfection efficiency.^21^ qRT-PCR results demonstrated that both si-ZIC5 #1 and si-ZIC5 #2 significantly reduced ZIC5 mRNA expression levels compared to the NC-si group (Figure 2b). Western blot analysis further confirmed a significant decrease in ZIC5 protein levels in A549 and H1299 cells (Figure 2c). These results collectively indicate that siRNA effectively inhibited ZIC5 expression in LUAD cells.

### Silencing ZIC5 significantly inhibits the proliferation of LUAD cells

**Figure 3.**
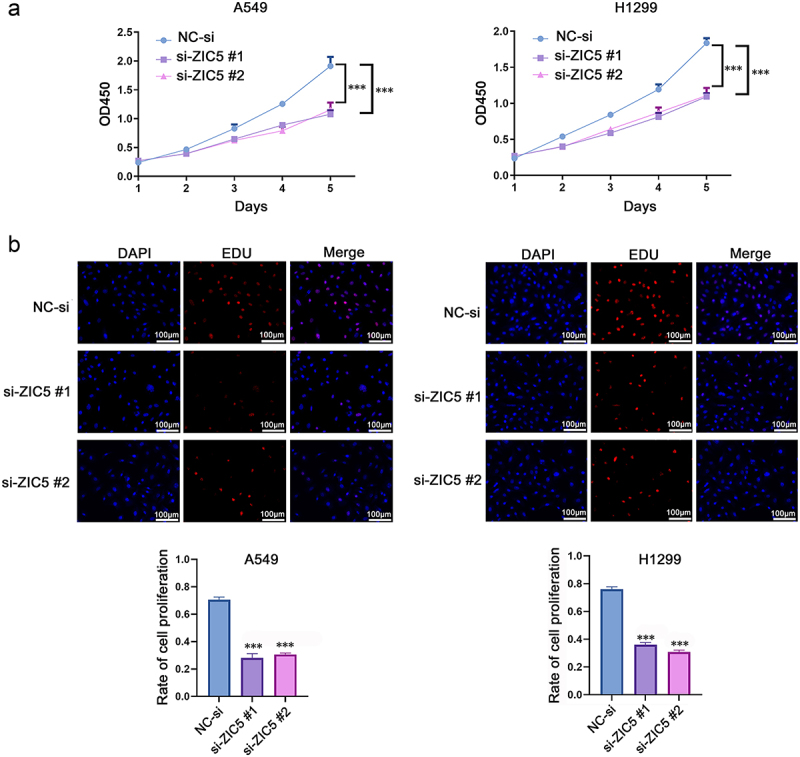
Silencing ZIC5 significantly inhibits the proliferation of LUAD cells. (a) CCK-8 assay measuring the proliferation activity of A549 and H1299 cells. (b) EdU incorporation assay detecting cell proliferation. EdU: 5-ethynyl-2’-deoxyuridine; DAPI: 4’,6-diamidino-2-phenylindole. Compared to the NC-si group, ***p < .001.

CCK-8 assay results showed that silencing ZIC5 significantly reduced the proliferation ability of A549 and H1299 cells, as evidenced by the OD450 values, which were significantly lower over time compared to the NC-si group (Figure 3a). EdU assays, which detect proliferating cells undergoing DNA replication, revealed that EdU, a thymidine analog that incorporates into newly synthesized DNA strands, was less incorporated in ZIC5-silenced cells compared to the NC-si group. This indicates that DNA synthesis was inhibited (Figure 3b). These results collectively suggest that silencing ZIC5 expression significantly inhibited the proliferation capacity of LUAD cells.

### Silencing ZIC5 significantly inhibits glycolytic function in LUAD cells

**Figure 4.**
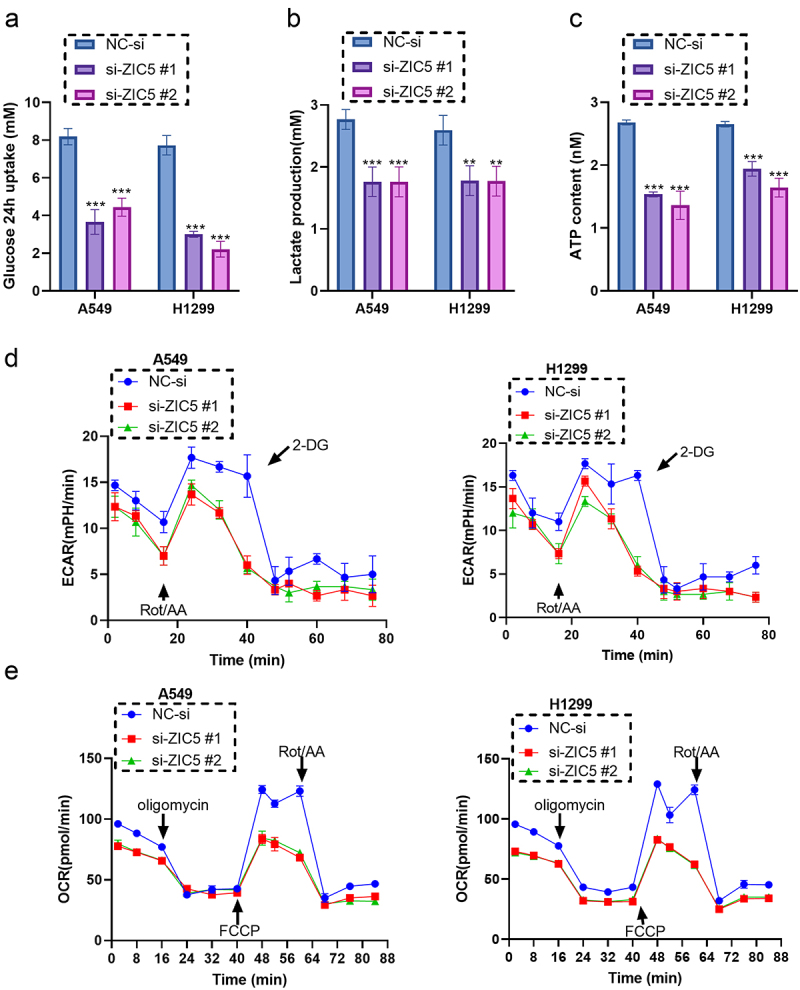
Silencing ZIC5 significantly inhibits glycolytic function in LUAD cells. (a) Measurement of 24 h glucose uptake. (b) Measurement of lactate production. (c) Measurement of ATP content, results expressed in mM. (d) Measurement of ECAR using the seahorse XF analyzer, with real-time monitoring by adding 2-DG, Rot/AA, and other reagents. (e) Measurement of OCR using the seahorse XF analyzer, with real-time monitoring by adding oligomycin, FCCP, Rot/AA, and other reagents. ECAR: extracellular acidification rate; OCR: oxygen consumption rate; 2-DG: 2-deoxy-D-glucose; Rot/AA: rotenone/antimycin A; FCCP: trifluoromethoxy carbonylcyanide phenylhydrazone. Compared to the NC-si group, **p < .01, ***p < .001.

After silencing ZIC5 expression, compared to the NC-si group, the 24 h glucose uptake of A549 and H1299 cells significantly decreased (Figure 4a). Lactate production also significantly decreased in both A549 cells and H1299 cells (Figure 4b). Additionally, ATP content significantly decreased (Figure 4c), indicating an inhibition of cellular energy metabolism. The ECAR was measured using the Seahorse XF Analyzer by adding 2-DG, Rot/AA, oligomycin, and FCCP at different time points. Results showed a significant decrease in ECAR following ZIC5 silencing (Figure 4d), and OCR also showed a significant decrease (Figure 4e). These results suggest that ZIC5 plays a crucial role in the energy metabolism of LUAD cells, with its absence leading to a significant reduction in both glycolysis and mitochondrial respiration functions.

### Silencing ZIC5 promotes disulfidptosis in LUAD cells

**Figure 5.**
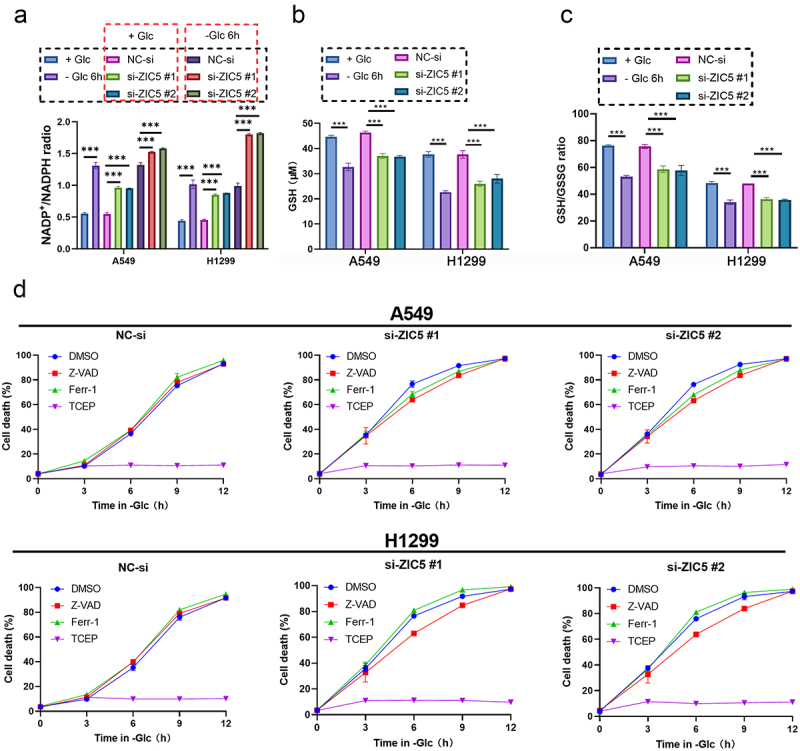
Silencing ZIC5 promotes disulfidptosis in LUAD cells. (a) Measurement of NADP^+^/NADPH ratio in cells. (b) Measurement of GSH content in cells. (c) Measurement of GSSG/GSH ratio in cells. (d) Measurement of cell death rate under glucose deprivation conditions following siRNA-mediated ZIC5 silencing with different treatments (DMSO, Z-VAD, ferrostatin-1, TCEP). Glc+: glucose repletion group; Glc-: glucose depletion group; NADP^+^: nicotinamide adenine dinucleotide phosphate (oxidized form); NADPH: nicotinamide adenine dinucleotide phosphate (reduced form); GSH: reduced glutathione; GSSG: oxidized glutathione; DMSO: dimethyl sulfoxide; Z-VAD: pan-caspase inhibitor; ferr-1: ferroptosis inhibitor; TCEP: tris(2-carboxyethyl) phosphine. Compared to the NC-si group, ***p < .001.

Compared to the glucose repletion (Glc+) group, the glucose depletion (Glc-) group showed a significant increase in NADP^+^/NADPH ratio (Figure 5a), a significant decrease in GSH content (Figure 5b), and a significant reduction in the GSH/GSSG ratio (Figure 5c). Following ZIC5 silencing, both A549 and H1299 cells exhibited a significant increase in NADP^+^/NADPH ratio (Figure 5a), a significant decrease in GSH content (Figure 5b), and a significant reduction in the GSH/GSSG ratio (Figure 5c) compared to the NC-si group. Under glucose depletion conditions, the increase in NADP^+^/NADPH ratio was more pronounced, indicating that glucose deprivation sensitizes cells to ZIC5 silencing-induced oxidative stress. In contrast, under glucose repletion conditions, ZIC5 silencing still resulted in an increase in the NADP^+^/NADPH ratio, but the effects were less marked than under glucose depletion, suggesting that glucose availability modulates the cellular response to ZIC5 inhibition (Figure 5a).

Under glucose deprivation conditions, the cell death rate in different treatment groups (DMSO, Z-VAD, Ferrostatin-1, TCEP) was measured. Compared to the TCEP treatment group, the cell death rate significantly increased following ZIC5 silencing. Z-VAD, an apoptosis inhibitor, showed a decrease in cell death rate following ZIC5 silencing compared to the DMSO group. TCEP, a disulfide stress inhibitor, showed a significant increase in cell death rate in the Z-VAD treatment group compared to the TCEP treatment group. Ferrostatin-1, a ferroptosis inhibitor, resulted in a lower cell death rate in the Ferrostatin-1 group than in the DMSO group for A549 cells, while the opposite was observed in H1299 cells. Compared to the TCEP treatment group, the Z-VAD treatment group showed a significant increase in cell death rate (Figure 5d). This suggests that the disulfide stress inhibitor TCEP can inhibit cell death in LUAD cells.

**Figure 6.**
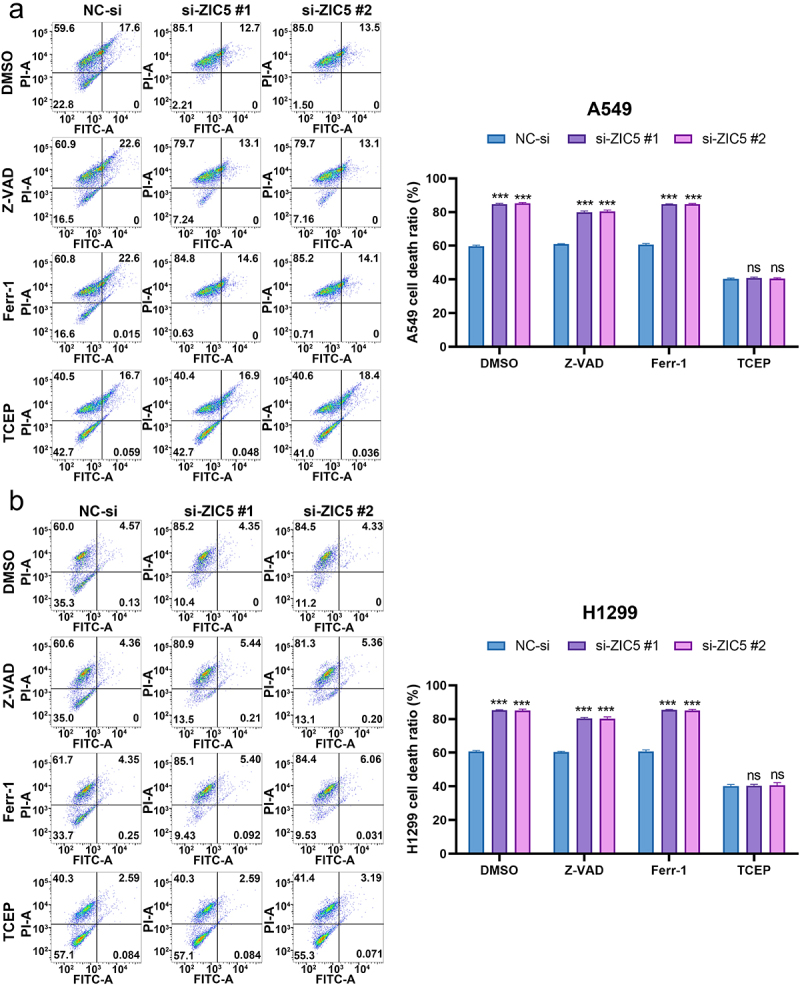
Silencing of ZIC5 significantly increases LUAD cells mortality by inducing apoptosis and ferroptosis. (a) Flow cytometry was used to assess apoptosis and cell death in A549 cells. (b) Flow cytometry was used to assess apoptosis and cell death in H1299 cells. Compared to the NC-si group, ***p < .001.

Flow cytometry results showed that under treatment with DMSO, Z-VAD, Ferr-1, and TCEP, the ZIC5 knockdown groups (si-ZIC5 #1 and si-ZIC5 #2) exhibited significantly higher cell death rates compared to the NC-si group (Figure 6a). For H1299 cells, ZIC5 knockdown also resulted in a higher cell death rate. Under treatment with DMSO, Z-VAD, Ferr-1, and TCEP, the si-ZIC5 groups showed significantly higher cell death rates than the NC-si group (Figure 6b).

**Figure 7.**
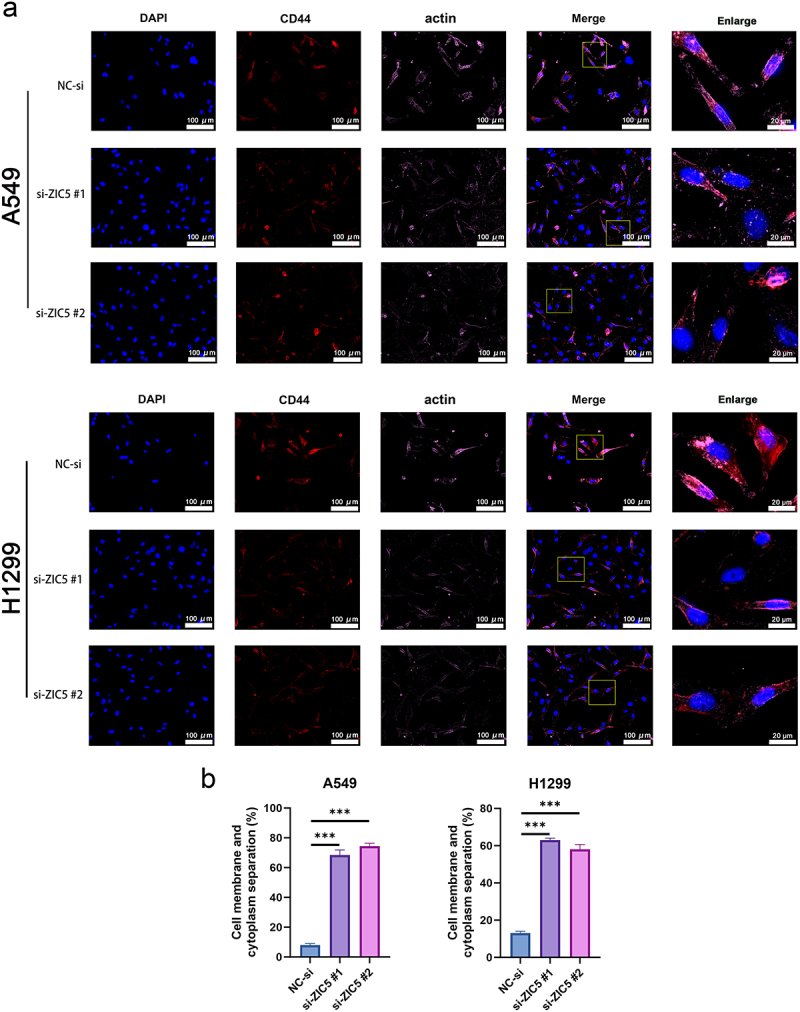
(a) immunofluorescence staining of CD44 and actin to observe the classic morphology of disulfidptosis. Merge: 400×, enlarge: 1500×. (b) Quantitative separation of cell membrane and cytoplasm (%).CD44: cluster of differentiation 44. Compared to the NC-si group, ***p < .001.

In immunofluorescence staining, CD44 was used to mark the cell membrane and actin to mark the cytoskeleton. Compared to the NC-si group, ZIC5 silencing resulted in a significant increase in CD44 expression, indicated by enhanced red fluorescence intensity in cells. Actin expression also significantly increased, shown by the enhanced purple fluorescence intensity in cells. The classic morphology of disulfidptosis, such as cell membrane rupture and separation of the cell membrane from the cytoplasm, was observed in the enlarged images (Figure 7). These results suggest that silencing ZIC5 significantly promotes disulfidptosis in LUAD cells by affecting redox status and related protein expression.

## Discussion

LUAD ranks as one of the predominant subtypes of lung cancer, with both incidence and mortality rates remaining high.^22^ Studies conducted by Sun et al. revealed that the expression of ZIC5 is significantly elevated in non-small cell lung cancer tissues compared to normal tissues, and silencing ZIC5 inhibited proliferation and metastasis of NSCLC cells.^23^ This study found that ZIC5 is overexpressed in LUAD, and such overexpression is significantly associated with poor prognosis for patients. Additionally, high expression of ZIC5 is positively correlated with the expression of glycolysis-related genes (such as HIF1A, EPO, and LDHA), suggesting that ZIC5 may play an important role in the glycolytic metabolism of LUAD. HIF1A (hypoxia-inducible factor 1α) is a key transcription factor that regulates tumor cell metabolism and helps tumor cells survive in hypoxic environments by activating the glycolytic pathway. Studies have shown that HIF1A expression is significantly upregulated in LUAD and is associated with poor prognosis.^24^ EPO (erythropoietin) regulates glycolysis and oxidative phosphorylation pathways in LUAD, promoting tumor growth and metastasis.^25^ LDHA (lactate dehydrogenase A) is a crucial enzyme in the glycolytic pathway, and its overexpression promotes lactate accumulation, leading to extracellular acidification, which enhances tumor invasiveness and metastatic potential.^26^ LDHA has also been linked to disulfidptosis, promoting lactate accumulation and triggering oxidative stress-induced cell death.^27^ Although HIF1A, EPO, and LDHA have been extensively studied in other cancers, research on these genes in LUAD remains limited. Furthermore, the role of LDHA in glycolysis and disulfidptosis provides a new perspective for this study. The hypoxia-inducible factor (HIF)-2α, or EPAS1, is a critical transcription factor involved in cellular responses to hypoxia. While both HIF-1α and EPAS1 regulate glycolysis and metastasis, HIF-1α governs the initial hypoxic response, whereas EPAS1 is activated under prolonged hypoxia, playing a role in mitochondrial metabolism and cell survival.^28^ The negative correlation between ZIC5 and EPAS1 expression may arise from their distinct regulatory roles. ZIC5 promotes tumor progression through metabolic pathways, possibly inhibiting EPAS1 activity or competing for the regulation of glycolytic genes. Additionally, EPAS1’s stability is greater than HIF-1α during prolonged hypoxia, which may further explain the inverse relationship between ZIC5 and EPAS1 in LUAD tissues.^29^

Furthermore, ZIC5 overexpression has been found to promote cell proliferation and invasion in multiple cancer cell types.^30,31^ GFP is a protein derived from jellyfish that can naturally emit green fluorescence and is widely used for monitoring gene expression and evaluating cell viability.^21^ Studies have shown that when GFP is transfected into cells, its fluorescence intensity is closely correlated with cellular metabolic activity (e.g., ATP levels) and the activity of gene expression.^21^ In this study, ZIC5 expression in the A549 and H1299 cell lines was silenced using siRNA technology. In si-ZIC5 cells, the GFP fluorescence intensity decreased and its distribution became uneven. The results suggest that after ZIC5 silencing, cell viability decreased and cellular stress increased, leading to a reduction in GFP expression and altered fluorescence distribution. Furthermore, the silencing of ZIC5 significantly inhibited the proliferation of A549 and H1299 cells, indicating that ZIC5 plays a crucial role in regulating cell proliferation.

Cancer cells often obtain energy rapidly through the glycolytic pathway, even under aerobic conditions, a phenomenon referred to as the Warburg effect.^32^ Lactate, the primary product of glycolysis, is indicative of the activity level of the intracellular glycolytic pathway, with its elevation commonly signaling increased glycolytic activity. High lactate levels also lead to extracellular acidification, which can influence the behavior of other cells in the tumor microenvironment, such as their invasive and migratory capabilities.^33^ The ATP content serves as a direct reflection of the cellular energy state. To meet the demands of rapid proliferation, cancer cells often require substantial amounts of ATP.^34^ This study found that after silencing ZIC5 expression, glucose uptake, lactate production, and ATP levels in A549 and H1299 cells were significantly reduced at 24 h, indicating that cellular energy metabolism was inhibited. ECAR reflects the cellular glycolytic activity, whereas OCR reflects mitochondrial respiratory function. By measuring these two parameters, the energy production ratio in different metabolic pathways can be distinguished.^35^ 2-DG, a glucose analogue, inhibits glycolysis through competitive inhibition of hexokinase, resulting in a significant decrease in cellular ATP levels.^36^ Moreover, the inhibitory effect of 2-DG on ECAR can directly reflect the glycolytic activity and metabolic state of the cells. Research shows that 2-DG is widely applied to study the contribution of the glycolytic pathway. By measuring the changes in ECAR before and after 2-DG treatment, the relative importance of glycolysis in cellular metabolism can be accurately assessed.^37^ Studies in breast cancer cells have shown that 2-DG significantly reduces both ECAR and OCR, indicating effective inhibition of glycolysis and mitochondrial respiration.^38^ By adding compounds such as 2-DG, Rotenone/antimycin A (Rot/AA), oligomycin, and carbonyl cyanide p-trifluoromethoxyphenylhydrazone (FCCP), glycolysis and mitochondrial respiration can be inhibited respectively, thereby dissecting the cellular responses under various metabolic conditions in detail. This study reveals that the silencing of ZIC5 leads to a significant reduction in both ECAR and OCR, indicating a critical role of ZIC5 in the energy metabolism of LUAD cells. Its absence results in a marked decrease in glycolysis and mitochondrial respiratory function, thus affecting cellular energy production and metabolic balance.

NADP^+^/NADPH and GSH play pivotal roles in maintaining the redox balance within cells.^39^ NADPH is primarily generated through the pentose phosphate pathway, providing reducing power for antioxidative systems, such as the reduction reaction of GSH reductase. Studies have indicated that insufficient production of NADPH elevates oxidative stress levels within cells, particularly in cancer cells. For instance, in LUAD, the upregulation of S100A2 promotes glutamine metabolism, reduces the NADP/NADPH ratio, and increases the GSH/GSSG ratio, thereby enhancing the survival and metastatic ability of tumor cells.^40^ Disulfidptosis, a type of cell death initiated by disulfide bond formation, is closely associated with oxidative stress.^41^ GSH, as the primary intracellular antioxidant, protects cells from oxidative damage by neutralizing reactive oxygen species (ROS). However, in disulfidptosis, depletion of GSH leads to inactivation of GSH peroxidase 4 (GPX4), thus promoting intracellular lipid peroxidation and cell death.^42^ Consequently, GSH plays a critical role in maintaining cellular antioxidant capability and preventing disulfidptosis. In studies of disulfidptosis in cancer, NADP^+^/NADPH and GSH levels serve as crucial indicators for assessing the cellular redox state. Research has shown that disulfidptosis is closely related to the metabolic state of the cell, particularly changes in glycolytic pathways and mitochondrial function. By inhibiting glucose metabolism or disrupting NADPH production pathways, oxidative stress levels in cancer cells can be increased, thereby inducing disulfidptosis. The use of 2-DG significantly inhibits glycolysis, reduces NADPH production, and increases oxidative stress, ultimately promoting disulfidptosis.^43^ By modulating NADP^+^/NADPH and GSH levels, the sensitivity of cancer cells to oxidative stress can be effectively enhanced, promoting cancer cell death and thereby improving therapeutic outcomes.^44^ For example, combining immunotherapy and chemotherapy can enhance the effects of disulfidptosis through increased oxidative stress, improving the cytotoxicity toward tumor cells.^42^ In this study, irrespective of the glucose conditions, silencing of ZIC5 significantly affects the redox balance and GSH metabolic status of cells. This suggests that ZIC5 plays a key role in regulating the redox balance and GSH metabolism in LUAD cells, where its silencing leads to decreased antioxidant capability and increased oxidative stress, thus promoting the occurrence of disulfidptosis.

Although we validated the function of ZIC5 in LUAD cells through in vitro cell experiments, the lack of validation using in vivo animal models remains a limitation. Future research should further investigate the role of ZIC5 in LUAD using animal models. Secondly, we discovered that ZIC5 is closely associated with the glycolytic pathway; however, its specific regulatory mechanisms are not yet clear. Further studies are required to elucidate how ZIC5 affects the expression and activity of glycolytic genes. Moreover, whether ZIC5 interacts with other signaling pathways to regulate LUAD progression is also an important direction for future research. Additionally, although CD44 was used as a membrane marker in this study, its role in regulating disulfidptosis was not the primary focus. The expression of CD44 on the cell membrane aligns with the observation of membrane and cytoplasm separation. However, the use of CD44 as a membrane marker may limit the interpretation of disulfidptosis, and alternative membrane markers were not utilized for further validation in this study.

In conclusion, ZIC5 plays a crucial role in promoting LUAD cell proliferation and energy metabolism while inhibiting disulfidptosis. Silencing ZIC5 markedly suppresses these processes, indicating its potential as a therapeutic target in LUAD.

## Data Availability

The data that support the findings of this study are available from the corresponding author upon reasonable request.
